# Association of an Algorithm‐Generated Medication Optimization Score With Clinical Outcomes in Ambulatory Patients With Heart Failure

**DOI:** 10.1002/phar.70101

**Published:** 2026-01-18

**Authors:** Mohamed S. Ali, Kaitlyn M. Greer, Sabah Ganai, Todd M. Koelling, Scott L. Hummel, Michael P. Dorsch

**Affiliations:** ^1^ College of Pharmac University of Michigan Ann Arbor Michigan USA; ^2^ Medical School University of Michigan Ann Arbor Michigan USA; ^3^ Frankel Cardiovascular Center University of Michigan Ann Arbor Michigan USA; ^4^ VA Ann Arbor Health System Ann Arbor Michigan USA

**Keywords:** advanced heart failure care, guideline‐directed medical therapy, heart failure with reduced ejection fraction, medication optimization score

## Abstract

**Aims:**

Guideline‐directed medical therapy (GDMT) implementation in heart failure with reduced ejection fraction (HFrEF) remains suboptimal. A computable algorithm was developed to generate a medication optimization score (MOS) and provide guideline‐based recommendations. This computable algorithm was previously validated using clinical trial data, and an updated version was developed in 2021 to include sodium‐glucose co‐transporter 2 inhibitors. This study evaluated the association between the medication optimization information generated by this version of the algorithm and clinical outcomes using real‐world data.

**Methods:**

We conducted a retrospective cohort study of 1352 ambulatory adult patients with chronic HFrEF who received care from the advanced heart failure service at the University of Michigan between July 1, 2021, and October 14, 2024. The algorithm‐generated MOS was calculated using electronic health record data. The primary outcome was a composite of all‐cause mortality or hospitalization. Cox proportional hazards models were used to evaluate the association between baseline MOS and the primary outcome. A time‐varying Cox model using the running cumulative MOS and a marginal structural model (MSM) was also conducted. A linear mixed‐effects model was used to assess improvement in MOS over time as the secondary outcome.

**Results:**

In the analysis adjusted for HF severity and comorbidities, baseline MOS was associated with a lower hazard of the composite outcome (hazard ratio (HR) 0.96, 95% confidence interval (95% CI): 0.92, 0.99, *p* = 0.040). In the cumulative time‐varying Cox model and the marginal structural model, the association with time‐varying MOS became stronger, with HRs of 0.88 (95% CI 0.81–0.95; *p* = 0.0015) and 0.88 (95% CI 0.83–0.93; *p* < 0.001), respectively. The event rates per 100 person‐years were 44.1 in MOS 0%–33%, 39.5 in MOS 34%–66%, and 31.8 in MOS 67%–100%. Longitudinally, MOS improved over time.

**Conclusion:**

Higher algorithm‐generated MOS values were significantly associated with lower all‐cause mortality or hospitalization, and the MOS values increased over the follow‐up period. This suggested that this algorithm effectively identifies opportunities for GDMT optimization in real‐world clinical settings.

## 1 Introduction

Heart failure (HF) affects an estimated 64 million people worldwide, with a significant 75% mortality rate within 5 years of the index HF hospitalization [1]. Clinical trials have established the benefit of using multiple disease‐modifying therapies for treating HF with reduced ejection fraction (HFrEF) [2, 3, 4, 5, 6, 7, 8, 9, 10, 11, 12]. The guideline and national performance and quality measures endorse the rapid implementation of these medications as they are proven to prolong survival without worsening HF events [13, 14]. Guidelines and these performance and quality measures also recommend target doses for each medication, as tolerated, based on doses tested in these clinical trials. Despite this, it is well‐documented that implementing these medications in their target doses is underutilized in clinical practice and can take years [15].

The American College of Cardiology (ACC) released an expert consensus decision pathway for optimizing HF management to address these issues [16]. This pathway recommends using electronic health records (EHRs) to enhance clinical decision‐making, reduce errors, and improve adherence to evidence‐based guidelines. In response, the pharmacy informatics group at the University of Michigan developed a computable algorithm based on the ACC/American Heart Association HfrEF guidelines [17]. This algorithm generates medication recommendations based on patients' clinical information and assigns patients a medication optimization score (MOS) based on their level of guideline‐directed medical therapy (GDMT) optimization. This algorithm was previously tested [17] using the GUIDE‐IT (Guiding Evidence‐Based Therapy Using Biomarker Intensified Treatment in Heart Failure) [18] and HF‐ACTION (Heart Failure: A Controlled Trial Investigating Outcomes of Exercise Training) [19] clinical trial data acquired from the NHLBI BioLINCC (National Heart Lung and Blood Institute, Biological Specimen and Data Repository Information Coordinating Center) repository. This algorithm was updated in 2021 to include sodium‐glucose co‐transporter 2 inhibitors (SGLT2i) in version 0.500. The objective of this study is to evaluate the association between the medication optimization information generated by this version of the algorithm and clinical outcomes using real‐world health system data.

## 2 Methods

### 2.1 Patient Population

This retrospective cohort study included adult ambulatory patients with chronic HFrEF at the University of Michigan advanced HF care program between July 1, 2021, and October 14, 2024. The advanced HF care program does not follow predefined referral criteria, and referrals are determined through clinical adjudication. The index date was the first clinical encounter with the advanced HF care team during the study period. Patients were identified from the EHRs if they were followed by the advanced HF team and diagnosed with HFrEF with left ventricular ejection fraction (LVEF) ≤ 40%. Exclusion criteria included patients who had undergone a heart transplant, those with an implanted ventricular support device, individuals who entered the cohort with fully optimized GDMT, and those with missing data for the Heart Failure Patient Severity Index (HFPSI). The University of Michigan Institutional Review Board approved this study.

### 2.2 Exposure of Interest

The primary exposure of interest was the MOS generated from the algorithm [17]. The MOS is a percentage ranging from 0% (least optimized) to 100% (most optimized), indicating the degree to which medication optimization needs to be performed. As the patient is prescribed more GDMT and at higher doses, the score approaches 100%. The MOS was calculated for all patients who met our inclusion criteria on the index date.

The current version of the algorithm (version 0.500) is configured to accept inputs including medication details (name and daily dose), New York Heart Association (NYHA) class, systolic blood pressure (SBP), heart rate (HR), serum creatinine levels (SCR), potassium levels, allergy or intolerance information, and race, focusing on one patient encounter per computation. The algorithm is being used in a patient‐centered clinical decision support system to help activate patient engagement in medication optimization in HFrEF and in a provider‐centered clinical decision support dashboard at the University of Michigan Health System.

When applied to clinical data, the algorithm uses this information to generate an MOS based on each patient's current medication regimen, laboratory values, and vital signs, and to provide recommendations for therapy adjustment. In this study, the algorithm was applied retrospectively to EHR data, producing both MOS and recommendations; however, the MOS served as the exposure of interest in this analysis.

### 2.3 Clinical Outcomes

The primary end point was the time from the index date to the first event of all‐cause mortality or hospitalization (hospital stay ≥ 1 day). This composite outcome was chosen because GDMT optimization reduces both hospitalization and mortality. The secondary end point was the change in the MOS across the study period. If the algorithm‐generated MOS is associated with the primary end point and can capture the improvement of GDMT optimization over time, this suggests its ability to identify patients with HFrEF with an opportunity for GDMT optimization in a real‐world setting.

### 2.4 HF Patient Severity Index (HFPSI) and Charlson Comorbidity Index (CCI)

Our primary end point analysis used the HFPSI risk stratification model to account for HF severity and the Charlson Comorbidity Index (CCI) to account for overall comorbidities. HFPSI includes blood urea nitrogen (BUN), B‐type natriuretic peptide (BNP), NYHA class, diabetes status, history of atrial fibrillation/flutter, and all‐cause hospitalization within the prior 6 months [20]. An integer HFPSI score based on the relative importance of each variable to the final HFPSI model was used, with one indicating the lowest level of HF severity and four indicating the highest. HFPSI is associated with the risk of death and/or all‐cause medical hospitalization in HF clinic outpatients [20].

The CCI is a validated tool that estimates the burden of comorbid diseases by assigning weighted scores to various chronic conditions (e.g., diabetes, renal disease, cancer) [21]. A higher CCI score indicates greater overall morbidity, which can be associated with poorer clinical outcomes [21]. Based on the CCI score, the severity of comorbidity was categorized into three grades: mild, with CCI scores of 1–2; moderate, with CCI scores of 3–4; and severe, with CCI scores ≥ 5 [22].

### 2.5 Statistical Analysis

Descriptive statistics were used to describe baseline patients' characteristics. Continuous variables were summarized as means ± standard deviations (SD), and categorical variables were summarized as frequencies (percentages). The baseline MOS was divided into tertiles based on the 33rd and 67th percentiles to compare baseline characteristics across the study cohort. This categorization was mainly for descriptive purposes and to build the Kaplan–Meier (KM) curve. However, our primary analysis used MOS as a continuous variable, scaled by 10‐unit increments, to detect the association at a finer scale.

The probability of event rates was visualized using the KM curve and compared across the baseline MOS tertiles using the log‐rank test for the primary outcome. Cox proportional hazards regression was used to estimate the association between the MOS and the hazard of the primary outcome. First, an unadjusted model was built to assess the unadjusted effect of the MOS at baseline on the time‐to‐event outcome. Second, an adjusted model was fitted, adjusting for demographics and HFPSI categories to account for the HF severity and CCI categories to account for the overall comorbidities. These two measures were selected for adjustment, rather than other HF measures and comorbidities, because they do not include variables represented in the algorithm. The proportional hazards assumption was tested using Schoenfeld residuals.

Third, the association between longitudinal changes in MOS and the primary outcome was examined using a time‐varying Cox proportional hazards model with the running cumulative average of each patient's MOS and a marginal structural model. Because patients varied in the frequency and timing of their clinical encounters, all encounters occurring within a 6‐month period were grouped into a single interval to standardize time points across the cohort. Six‐month intervals were chosen as clinically meaningful because, in HF care, medication adjustments commonly occur every few months, and 6‐month intervals allow sufficient time for titration of therapy and meaningful changes in the MOS. As a result, the follow‐up period was divided into seven periods: baseline (Time 0), 0–6 months (Time 1), 6–12 months (Time 2), 12–18 months (Time 3), 18–24 months (Time 4), 24–30 months (Time 5), 30–36 months (Time 6), and 36–42 months (Time 7). Because each patient had multiple encounters within each time interval, the average MOS for each patient was calculated over that time interval.

For the cumulative time‐varying Cox proportional hazard model, the time‐varying exposure for each 6‐month interval was the 1‐interval‐lagged cumulative mean of MOS, computed from all MOS values observed prior to the start of that interval. We adjusted for the baseline value of the covariates (age, sex, race, ethnicity, CCI, HFPSI) and used patient‐clustered robust standard errors.

The marginal structural model was fit to allow for MOS to be considered as a time‐varying exposure while also accounting for prior MOS scores in the presence of time‐varying confounders (HFPSI and CCI). The marginal structural model used inverse probability of treatment weighting (IPTW) and inverse probability of censoring weighting (IPCW). Since the exposure variable (MOS) was modeled as a continuous variable, scaled per 10‐unit increase, the IPTW was derived from stabilized weights based on the ratio of conditional probability densities from the exposure models. Time‐varying confounders included HFPSI and CCI, and baseline covariates included age, sex, race, and ethnicity. Specifically, the numerator model included the prior MOS, baseline covariates, and time, and the denominator model additionally included time‐varying covariates. Because encounters were aggregated into 6‐month intervals and within‐interval ordering could not be guaranteed, time‐varying confounders (HFPSI, CCI) were lagged by one interval in the weight models to ensure conditioning only on information available prior to the exposure. The IPCW was estimated to account for differential loss to follow‐up, using logistic regression models for censoring at each time point. Censoring was defined as the end of follow‐up for a patient without experiencing the outcome event. The numerator IPCW model included prior MOS, baseline covariates, time, and baseline distance from the University of Michigan; the denominator model additionally included lagged time‐varying covariates (HFPSI and CCI). The total stabilized weight for each observation was defined as the product of the truncated IPTW and IPCW (1st–99th percentile truncation). The final MSM was fit using a weighted Cox proportional hazards model. Robust standard errors were computed using clustering by patient identifier to account for within‐subject correlation.

For the secondary outcome, longitudinal trends in MOS were analyzed from the index date through the follow‐up period to assess changes in the MOS over time. Then, these patient‐level MOS averages were summarized across the cohort using the mean, standard deviation, 95% confidence interval (CI), median, and interquartile range (IQR). Longitudinal changes in the MOS were tested using a linear mixed‐effects model to account for repeated measurements within patients. Time since baseline (in quarters) was modeled as a continuous variable to estimate the average change in MOS per quarter, with random intercepts and random slopes specified for each patient to allow individual variability in both baseline MOS and rate of change to partly account for unbalanced follow up. The improvement in model fit from adding random slopes was assessed with a likelihood ratio test comparing the random‐intercept–only and random‐slope models. The analysis was conducted after excluding patients with MOS = 100% at baseline. The cohort comprised 1751 patients for this secondary end point analysis. All statistical analyses were conducted using R programming (Version 4.4.0; R Foundation for Statistical Computing, Vienna, Austria).

## 3 Results

### 3.1 Patient Characteristics

**FIGURE 1:**
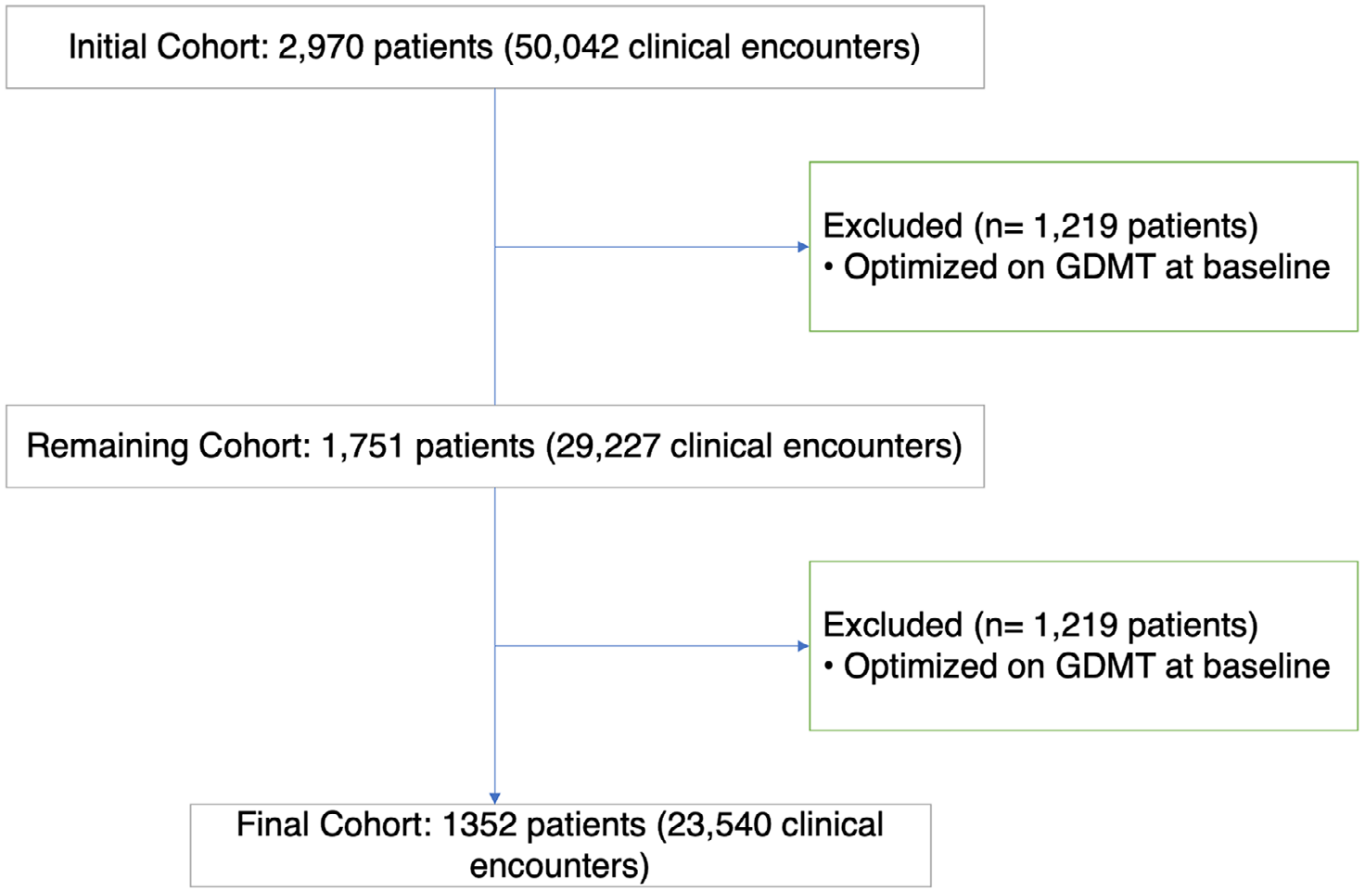
Flowchart depicting the inclusion and exclusion criteria for the study cohort. HFPSI, heart failure patient severity index; GDMT, guideline‐directed medical therapy.

The initial cohort included 2970 patients with a total of 50,042 clinical encounters. After excluding 1219 patients who were fully optimized on GDMT at baseline as measured by the MOS, the cohort was reduced to 1751 patients with 29,227 clinical encounters. Additionally, we excluded patients who did not have information about HF severity as measured by HFPSI. The final study cohort comprised 1352 patients with 23,540 clinical encounters. Figure 1 depicts the selection of patients included in the analysis.

The mean age was 64.7 ± 14.3 years, and 65.0% were male. White patients comprised 75.5% of the cohort, while 19.0% were African American and 2.4% Hispanic or Latino. The mean systolic blood pressure (SBP) and diastolic blood pressures were 121.0 ± 17.3 mmHg and 68.8 ± 12.1 mmHg, respectively. Mean serum creatinine (SCR) was 1.33 ± 0.94 mg/dL, potassium was 4.4 ± 0.45 mmol/L, and Estimated Glomerular Filtration Rate (eGFR) was 63.0 ± 23.0 mL/min/1.73 m^2^. The mean left ventricular ejection fraction (LVEF) was 28.9% ± 8.1%.

Patients were categorized into baseline MOS tertiles: T1 (MOS 0%–33%), T2 (MOS 34%–67%), and T3 (MOS 68%–100%), with mean MOS scores of 34.36% ± 12.66% in T1, 61.70% ± 4.24% in T2, and 79.39% ± 7.98% in T3. Patients in T3 were younger (mean age 61.8 vs. 67.2 years in T1) and more likely to be classified as HFPSI class 1 (60.3% vs. 40.1% in T1). NYHA class IV was less common in T3 (1.2%) compared to T1 (3.8%). Additional descriptive statistics are shown in Table 1, including distributions across MOS categories. Table S1 shows target dose achievement by medication class and full optimization at end of follow‐up.

**TABLE 1:** Patient characteristics.

Characteristic	All patients	MOS tertiles		
	All tertiles	T1 (0%–33%)	T2 (34%–67%)	T3 (68%–100%)
*n*	1352	549	392	411
Mean MOS, %	56.00 ± 21.00	34.36 ± 12.66	61.70 ± 4.24	79.39 ± 7.98
Age in years, mean (SD)	64.72 (14.34)	67.22 (14.49)	64.27 (14.34)	61.82 (13.59)
*Sex, n (%)*				
Female	473 (35.0)	216 (39.3)	124 (31.6)	133 (32.4)
Male	879 (65.0)	333 (60.7)	268 (68.4)	278 (67.6)
*Race, n (%)*				
White	1021 (75.5)	420 (76.5)	306 (78.1)	295 (71.8)
African American	257 (19.0)	100 (18.2)	63 (16.1)	94 (22.9)
Other	74 (5.5)	29 (5.3)	23 (5.9)	22 (5.4)
*Ethnicity, n (%)*				
Hispanic or Latino	32 (2.4)	15 (2.7)	6 (1.5)	11 (2.7)
Non‐Hispanic or Latino	1293 (95.6)	519 (94.5)	380 (97.4)	394 (95.9)
Unknown/Refused	27 (2.0)	15 (2.7)	6 (1.5)	6 (1.5)
Systolic Blood Pressure in mm Hg, mean (SD)	121.00 (17.3)	123.34 (17.46)	115.61 (17.76)	124.39 (15.04)
Diastolic Blood Pressure in mm Hg, mean (SD)	68.80 (12.10)	69.11 (12.75)	66.60 (12.05)	70.38 (10.97)
Pulse (bpm), mean (SD)	76.50 (13.80)	77.88 (14.03)	76.27 (13.73)	75.04 (13.49)
Serum Creatinine, mean (SD)	1.33 (0.94)	1.33 (0.83)	1.33 (0.98)	1.32 (1.04)
LVEF, mean (SD)	28.90 (8.12)	29.65 (7.96)	27.97 (8.42)	28.90 (7.95)
eGFR, mean (SD)	62.66 (23.06)	59.59 (23.20)	63.26 (23.70)	66.20 (21.72)
Serum K+ level, mean (SD)	4.396 (0.4509)	4.36 (0.44)	4.44 (0.49)	4.41 (0.42)
*HFPSI, n (%)*				
1	628 (46.4)	220 (40.1)	160 (40.8)	248 (60.3)
2	311 (23.0)	121 (22.0)	116 (29.6)	74 (18.0)
3	250 (18.5)	127 (23.1)	67 (17.1)	56 (13.6)
4	163 (12.1)	81 (14.8)	49 (12.5)	33 (8.0)
*CCI, n (%)*				
Mild	269 (19.9)	94 (17.1)	77 (19.6)	98 (23.8)
Moderate	284 (21.0)	106 (19.3)	81 (20.7)	97 (23.6)
Severe	799 (59.1)	349 (63.6)	234 (59.7)	216 (52.6)
*NYHA, n (%)*				
1	148 (10.9)	14 (2.6)	17 (4.3)	117 (28.5)
2	631 (46.7)	256 (46.6)	203 (51.8)	172 (41.8)
3	523 (38.7)	258 (47.0)	148 (37.8)	117 (28.5)
4	50 (3.7)	21 (3.8)	24 (6.1)	5 (1.2)
ACEIs/ARBs/ARNI, *n* (%)	1051 (77.7)	351 (63.9)	314 (80.1)	386 (93.9)
Beta‐blockers, *n* (%)	1134 (83.9)	419 (76.3)	335 (85.5)	380 (92.5)
MRA, *n* (%)	746 (55.2)	159 (29.0)	275 (70.2)	312 (76.0)
SGLT2is, *n* (%)	288 (21.3)	34 (6.2)	69 (17.6)	185 (45.0)

Abbreviations: ACEIs = angiotensin‐converting enzyme inhibitors, ARBs = angiotensin receptor blockers, ARNI = angiotensin receptor‐neprilysin inhibitors, bpm = beats per minute, CCI = Charlson comorbidity index, LVEF = left ventricular ejection fraction, eGFR = estimated glomerular filtration rate, HFPSI = heart failure patient severity index, MOS = medication optimization score, MRAs = mineralocorticoid receptor antagonists, NYHA = New York heart association classification, SD = standard deviation, SGLT2is = Sodium‐Glucose Cotransporter 2 inhibitors.

### 3.2 Medication Optimization Score and Clinical Outcomes

Over 1496 person‐years of follow‐up, there were 582 events, resulting in an overall event rate of 38.9 events per 100 person‐years. The MOS was significantly associated with a reduction in all‐cause mortality and hospitalization across all models. In the unadjusted model (model A), the hazard ratio (HR) for the MOS scaled per 10 units was 0.93 (95% CI: 0.90–0.97, *p* < 0.001). After adjusting for HFPSI and CCI in model B, this association remained significant yet slightly attenuated, with an HR of 0.96 (95% CI: 0.92–0.99, *p* = 0.040). In the Cox model with the running‐average MOS (model C), the HR for the MOS scaled per 10 units was 0.88 (95% CI: 0.81–0.95; *p* = 0.001). In MSM model (model D), the HR for the MOS scaled per 10 units was 0.88 (95% CI: 0.83–0.93; *p* < 0.001). These results are summarized in Table 2.

**TABLE 2:** Cox proportional hazard models.

Variable	Model A (HR [95% CI], *p*)	Model B (HR [95% CI], *p*)	Model C (HR [95% CI], *p*)	Model D (HR [95% CI], *p*)
MOS per 10 units	0.93 [0.90, 0.97], *p* < 0.001	0.96 [0.92, 0.99], *p* = 0.040	0.88 [0.81–0.95], *p* = 0.001	0.88 [0.83–0.93], *p* < 0.001
Age per 10 years	—	1.17 [1.09–1.25], *p* < 0.001	1.39 [1.21–1.60], *p* < 0.001	—
*Gender*				
Male	—	Ref	Ref	—
Female	—	0.94 [0.79, 1.11], *p* = 0.466	0.79 [0.56–1.10], *p* = 0.500	—
*Race*				
White African	—	Ref 1.17 [0.94, 1.45]	Ref 1.38 [0.89–2.14]	—
American	—	*p* = 0.152	*p* = 0.151	—
Other	—	1.22 [0.84, 1.76], *p* = 0.303	1.87 [0.98–3.59], *p* = 0.059	—
*Ethnicity*				
Non‐Hispanic or Latino	—	Ref	Ref	—
Hispanic or Latino	—	0.93 [0.52–1.65], *p* = 0.802	0.90 [0.33–2.45], *p* = 0.834	—
Unknown/Refused	—		1.34 [0.46–3.87], *p* = 0.587	—
*HFPSI*				
1	—	Ref	Ref	—
2	—	1.21 [0.98–1.50], *p* = 0.078	1.52 [0.95–2.43], *p* = 0.080	—
3	—	1.19 [0.93–1.52], *p* = 0.162	1.64 [1.01–2.64], *p* = 0.043	—
4	—	2.00 [1.54–2.59], *p* < 0.001	2.97 [1.88–4.69], *p* < 0.001	—
*CCI*				
Mild	—	Ref	Ref	—
Moderate	—	0.86 [0.64–1.16], *p* = 0.313	1.22 [0.54–2.76], *p* = 0.630	—
Severe	—	1.10 [0.86–1.43], *p* = 0.446	2.56 [1.28–5.15], *p* = 0.008	—

*Note:* Model A represents the unadjusted association between baseline MOS and the outcome, Model B adjusts for baseline demographic and clinical characteristics, Model C represents a time‐varying Cox proportional hazards model using the running (cumulative mean) MOS across follow‐up, adjusted for the same baseline covariates, Model D represents a marginal structural Cox model estimating the association between time‐varying MOS and the outcome, weighted by the product of inverse probability of treatment and censoring weights (IPTW × IPCW) to account for time‐varying confounding.

Abbreviations: CCI = Charleson comorbidity index, CI = confidence interval, HFPSI = heart failure patient severity index, HR = hazard ratio, MOS = medication optimization score.

**FIGURE 2:**
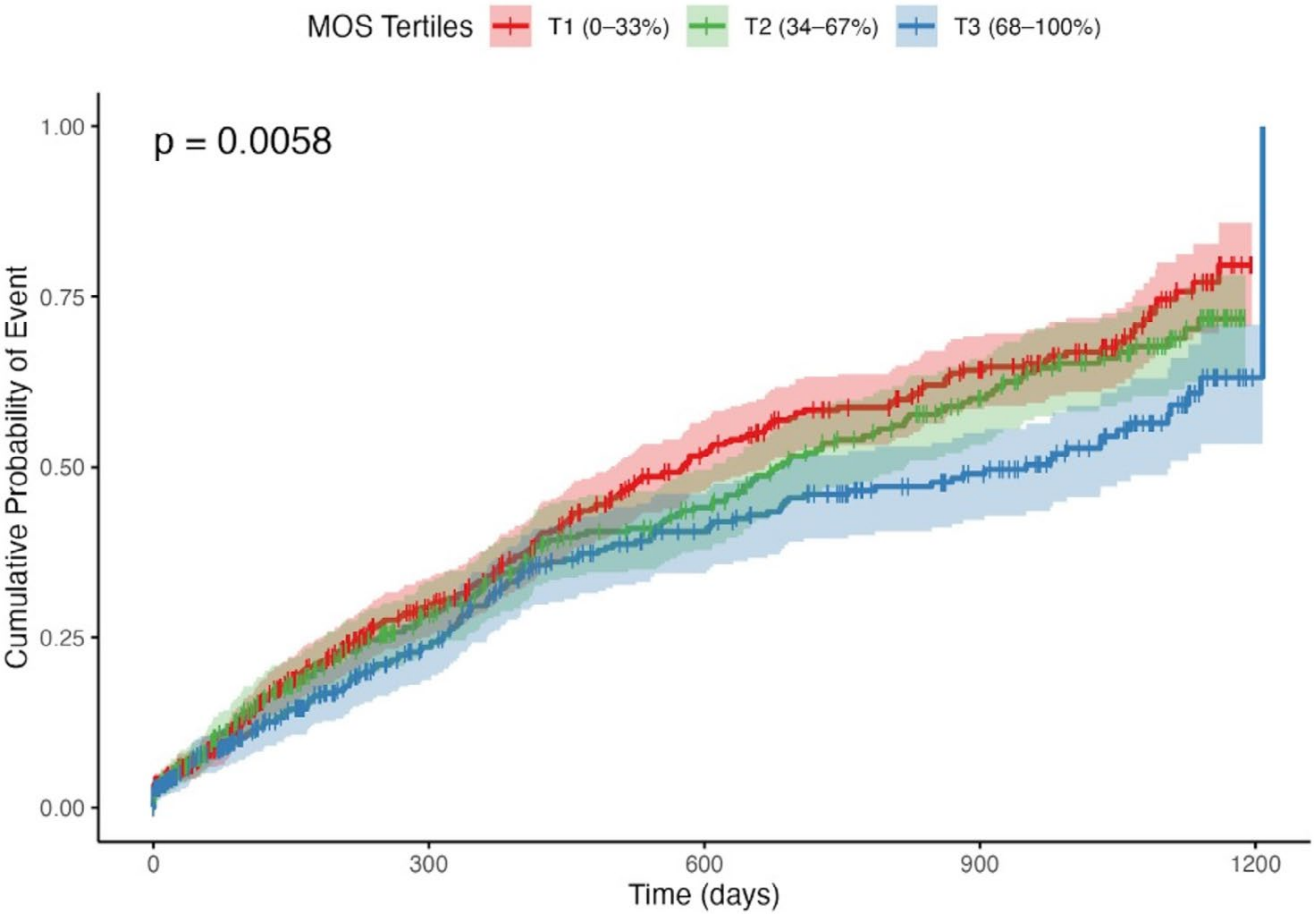
Cumulative Probability of Event by MOS Tertiles: Kaplan–Meier curves showing the cumulative probability of the primary event over time, stratified by MOS tertiles. Shaded areas represent 95% confidence intervals for the cumulative probabilities.

The event rates per 100 person‐years were 44.1 events per 100 person‐years in T1, 39.5 in T2, and 31.8 in T3. In the cumulative probability of the event curve stratified by the baseline MOS tertiles, as shown in Figure 2, there was a significant stepwise reduction in the cumulative probability of all‐cause mortality or hospitalization with increasing MOS tertiles (*p* = 0.0058). Patients in the highest tertile (T3: MOS 68%–100%) exhibited the lowest cumulative probability over time, followed by those in the middle tertile (T2: MOS 34%–67%), whereas the lowest tertile (T1: MOS 0%–33%) exhibited the highest cumulative probability of all‐cause mortality or hospitalization.

### 3.3 Medication Optimization Over Time

**FIGURE 3:**
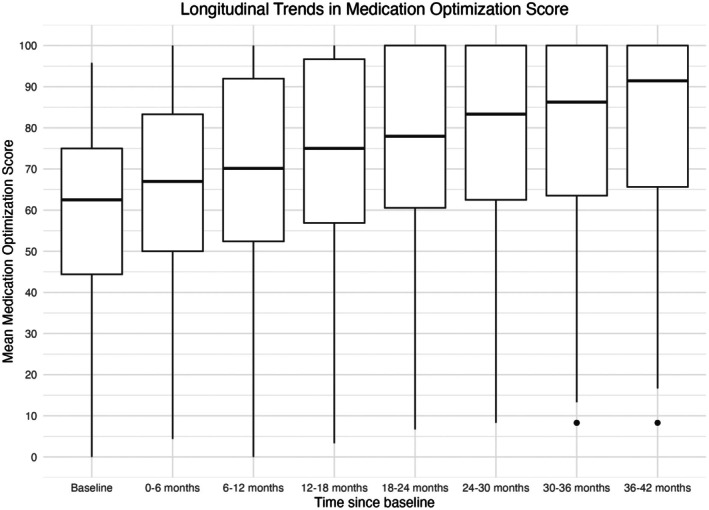
Longitudinal Trends in Medication Optimization Score. This figure displays the distribution of the MOS at baseline and across successive 6‐month intervals over a 42‐month follow‐up period.

At baseline (Time 0), the mean MOS was 59% (95% CI: 58%, 60%), and the median MOS was 62% (IQR: 44, 75%). Over the follow‐up period, there was a consistent upward trend in MOS. By 6–12 months (Time 2), the mean MOS increased to 70% (95% CI: 68, 71%), with a median of 70% (IQR: 52, 92%). This trend continued through the 36–42 month interval (Time 7), where the mean MOS reached 81% (95% CI: 78, 84%) and the median increased to 91% (IQR: 66, 100%). The longitudinal trend in the MOS across these sequential time intervals is visualized in Figure 3.

The longitudinal mixed‐effects model showed a significant positive trend in MOS over time. The mean baseline MOS was 61% (95% CI 60%–62%; *p* < 0.001), and MOS increased by an average of 3.3 percentage points (pp) per quarter (6 months) (95% CI: 3.00–3.70 pp; *p* < 0.001). Allowing for random slopes significantly improved model fit compared with a random‐intercept–only model (*p* < 0.001), indicating substantial variability in individual patient trajectories.

## 4 Discussion

In this study, the updated algorithm‐generated MOS was significantly associated with the risk of all‐cause mortality and/or hospitalization. The MOS also improved over the follow‐up period. Together, these findings suggest that the MOS effectively highlights opportunities for GDMT improvement and captures real‐world patterns of GDMT optimization.

The MOS integrates two key components: the number of GDMT drug classes prescribed and the degree of dose intensification. Both components are well‐established determinants of outcomes in HF. Prior clinical studies consistently show that patients receiving more GDMT classes and higher tolerated doses experience lower mortality and hospitalization rates. For example, a network meta‐analysis demonstrated that triple therapy with an angiotensin‐converting enzyme (ACE) inhibitor or angiotensin receptor blocker (ARB), β‐blocker, and mineralocorticoid receptor antagonist (MRA) reduces all‐cause mortality by approximately 48%, while adding angiotensin receptor‐neprilysin inhibitor (ARNI) and SGLT2 inhibitor achieves up to a 61% reduction compared with no GDMT [23]. Real‐world data support these findings. In one large cohort of patients with HFrEF, triple‐class therapy reduced 2‐year mortality by 29% relative to monotherapy, whereas patients receiving no GDMT had the highest mortality rate (21%) [24].

Dose intensity also matters. The ATLAS [10] and HEAAL [25] trials showed that higher doses of ACE inhibitors or ARBs significantly reduced HF hospitalizations compared with lower doses, and the HF‐ACTION study [26] found that de‐escalation of ACE inhibitors or β‐blockers was associated with increased mortality.

Consistent with prior observations, our study showed that MOS values tended to rise over time, reflecting a gradual improvement in GDMT use, though optimization remained incomplete. Observational data in newly diagnosed HFrEF populations demonstrated similar trends. GDMT use increases steadily over the first year (e.g., ACEi/ARB/ARNI use rising from approximately 14% at baseline to 65% at 12 months) yet few patients achieve target doses [27]. In the CHAMP‐HF registry, less than 1% reached target doses of all three major drug classes simultaneously [28]. Overall, the MOS mirrored these real‐world patterns: it correlated with clinical outcomes and captured the progressive yet incomplete optimization of GDMT in routine HF care.

Quantifying GDMT intensity is foundational for HF care. Thus, multiple methods have been developed to assess GDMT optimization in HF. One approach is the HF collaboratory (HFC) score, which was developed by a delegation of representatives from academia, industry, and government in 2019 [29]. In this scoring system, each drug class contributes points according to the proportion of the target dose a patient is prescribed, and these points are then summed to produce the overall HFC score. For beta‐blockers, ACEIs, and ARBs, point allocations are 0 for no treatment, 1 for doses below 50% of the target, and 2 for doses at or above 50%. Mineralocorticoid receptor antagonists and ARNIs receive 0 points if not prescribed, 2 points for any MRA dose, and 3 points for any ARNI dose. Accordingly, the possible HFC scores in this score range from 0 to 7. Any documented contraindication to a specific GDMT class was treated as guideline‐adherent therapy for that class. The HFC score has been modified and applied to the DAPA HF clinical trial patients. In this modified HFC score (mHFC), the total number of possible points ranges from 0 to 8. Each class is assigned points: 0 for no treatment, 1 for less than 50% of the target daily dose, and 2 for reaching or exceeding 50% of the target daily dose. The mHFC score allocates points separately for the sacubitril and valsartan components for ARNI therapy. Consequently, if a patient is on the target dosage of sacubitril/valsartan, that single medication yields 4 points (out of the maximum of 8 points). To convert the mHFC total into a 100‐point scale, the total score is divided by the maximum of 8 points and then multiplied by 100. As with the HFC score, documented contraindications for a GDMT class are treated as guideline adherence.

The Kansas City Medical Optimization (KCMO) score was developed to quantify GDMT use and intensity [30]. The KCMO score is an average proportion of prescribed‐to‐target doses for each eligible GDMT class. First, the total daily dose for each drug class is calculated by multiplying the prescribed dose by the frequency. The total daily dose of each eligible medication class is divided by its recommended target dose. These fractions are then averaged across all classes that the patient can receive. An exception is for MRAs, which in this score was assigned a value of either 1 (if any dose was prescribed) or 0 (if none was prescribed).

The MOS was developed in 2018 and updated in 2021 to include the SGLT2i class; the updated algorithm‐generated MOS is used and evaluated in this study [17]. In this score, the algorithm assesses whether the recommended target dose of each of the four medication classes has been reached, with fewer points awarded for partial doses. These points are tallied across all four classes, and the patient's actual total is then calculated, producing a final MOS value on a 0%–100% scale.

The MOS differs from prior GDMT scoring methods in several ways. First, unlike other algorithms, the MOS relies on patient‐specific clinical parameters—including blood pressure, HR, and relevant laboratory results—to assess eligibility for each medication class and dose escalation. This ensures that patients are not penalized for clinical ineligibility. Second, whereas both the HFC score and its modified version (mHFC) use threshold‐based categorizations (e.g., ≥ 50% versus < 50% of the target dose), the MOS utilizes fractional dosing increments. This more precisely captures clinically meaningful titrations that may fall below these threshold cutoffs, such as increasing a beta‐blocker from 25 to 50 mg, which may improve outcomes. Third, the MOS excludes medication classes from its denominator when a documented intolerance or allergy is present, rather than treating such cases as equivalent to receiving the full recommended dose. This minimizes the risk of artificially elevating a patient's overall score. Fourth, although the KCMO score likewise quantifies GDMT dosing in proportional terms, it incorporates MRAs in a binary fashion and provides no built‐in algorithm to facilitate medication adjustments. In contrast, the MOS is embedded in a rule‐based clinical decision‐support system that not only generates a percentage‐based score but also provides individualized recommendations (e.g., initiating an MRA or titrating an ACE inhibitor). Finally, the MOS algorithm can accommodate new guidelines without requiring substantial recalibration of point thresholds.

This study has some limitations. First, this analysis was retrospective and relied on data from select patient populations; the cohort was drawn from a single academic health system, which may not accurately reflect the broader HF population. Second, the analysis adjusted for HFPSI and CCI; however, these measures do not capture all relevant confounding variables, leaving the possibility of residual confounding. Third, relying on EHR data may introduce inaccuracies or incomplete documentation (e.g., medication adherence, contraindications, or intolerances not captured), which can influence both the MOS calculations and clinical outcome assessments. Fourth, if patients receive care outside our health system, those external encounters may not be reflected in our records, leading to potential underestimation of hospitalizations, medication changes, or clinical events that occurred elsewhere.

## 5 Conclusion

In this real‐world cohort of patients with HFrEF, the updated algorithm‐generated MOS was consistently associated with lower all‐cause mortality or hospitalization and effectively tracked incremental improvements in GDMT over time. This suggests its ability to identify opportunities to enhance GDMT in patients with HFrEF.

## Author Contributions

**Mohamed S. Ali:** writing – original draft. **Kaitlyn M. Greer:** writing – review and editing. **Sabah Ganai:** writing – review and editing. **Todd M. Koelling:** conceptualization, methodology, writing – review and editing. **Scott L. Hummel:** conceptualization, methodology, writing – review and editing. **Michael P. Dorsch:** conceptualization, methodology, writing – review and editing.

## Funding

The authors have nothing to report.

## Conflicts of Interest

The authors declare no conflicts of interest.

## Supporting information


**Table S1:** Target dose achievement by medication class and full optimization at end of follow‐up.

## Data Availability

The datasets generated during and/or analyzed during the current study may be available from the corresponding author upon reasonable request.
