# Supplementary material for: Association of an Algorithm‐Generated Medication Optimization Score With Clinical Outcomes in Ambulatory Patients With Heart Failure
Source: Pharmacotherapy. 2026 Jan 18;46(1):e70101. doi: 10.1002/phar.70101 (PMC12812323; doi:10.1002/phar.70101)
Supplement: Supplementary file 1 — Table S1: Target dose achievement by medication class and full optimization at end of follow‐up. [file PHAR-46-0-s001.docx]

Supplementary Table 1: Target dose achievement by medication class and full optimization at end of follow-up.

| Medication class / metric | n (%) |
| --- | --- |
| ACEI/ARB/ARNI target | 915 (67.7) |
| β-blocker target | 956 (70.7) |
| MRA target | 1,059 (78.3) |
| SGLT2 inhibitor target | 831 (61.5) |
| Hydralazine/isosorbide dinitrate target | 192 (14.2) |
| MOS = 100% (fully optimized) | 398 (29.4) |

ACEI = angiotensin-converting enzyme inhibitor; ARB = angiotensin receptor blocker; ARNI = angiotensin receptor–neprilysin inhibitor; MRA = mineralocorticoid receptor antagonist; MOS = Medication Optimization Score; SGLT2 = sodium-glucose co-transporter 2.
